# Free-breathing 3D Stack of Stars GRE (StarVIBE) sequence for detecting pulmonary nodules in ^18^F-FDG PET/MRI

**DOI:** 10.1186/s40658-022-00439-1

**Published:** 2022-02-07

**Authors:** Nils Martin Bruckmann, Julian Kirchner, Janna Morawitz, Lale Umutlu, Wolfgang P. Fendler, Ken Herrmann, Ann-Kathrin Bittner, Oliver Hoffmann, Tanja Fehm, Maike E. Lindemann, Christian Buchbender, Gerald Antoch, Lino M. Sawicki

**Affiliations:** 1 Department of Diagnostic and Interventional Radiology, Medical Faculty, University Dusseldorf, Moorenstrasse 5, 40225 Düsseldorf, Germany; 2 Department of Diagnostic and Interventional Radiology and Neuroradiology, University Hospital Essen, University of Duisburg-Essen, 45147 Essen, Germany; 3 Department of Nuclear Medicine, University Hospital Essen, University of Duisburg-Essen and German Cancer Consortium (DKTK), Essen, Germany; 4 Department Gynecology and Obstetrics, University Hospital Essen, University of Duisburg-Essen, 45147 Essen, Germany; 5 Department of Gynecology, Medical Faculty, University Dusseldorf, 40225 Düsseldorf, Germany; 6 High-Field and Hybrid MR Imaging, University Hospital Essen, University of Duisburg-Essen, 45147 Essen, Germany

**Keywords:** Lung nodule detection, PET/MRI, Breast cancer, Computed tomography

## Abstract

**Background:**

The free-breathing T1-weighted 3D Stack of Stars GRE (StarVIBE) MR sequence potentially reduces artifacts in chest MRI. The purpose of this study was to evaluate StarVIBE for the detection of pulmonary nodules in ^18^F-FDG PET/MRI.

**Material and methods:**

In this retrospective analysis, conducted on a prospective clinical trial cohort, 88 consecutive women with newly diagnosed breast cancer underwent both contrast-enhanced whole-body ^18^F-FDG PET/MRI and computed tomography (CT). Patients’ chests were examined on CT as well as on StarVIBE and conventional T1-weighted VIBE and T2-weighted HASTE MR sequences, with CT serving as the reference standard. Presence, size, and location of all detectable lung nodules were assessed. Wilcoxon test was applied to compare nodule features and Pearson’s, and Spearman’s correlation coefficients were calculated.

**Results:**

Out of 65 lung nodules detected in 36 patients with CT (3.7 ± 1.4 mm), StarVIBE was able to detect 31 (47.7%), VIBE 26 (40%) and HASTE 11 (16.8%), respectively. Overall, CT showed a significantly higher detectability than all MRI sequences combined (65 vs. 36, difference 44.6%, *p* < 0.001). The VIBE showed a significantly better detection rate than the HASTE (23.1%, *p* = 0.001). Detection rates between StarVIBE and VIBE did not significantly differ (7.7%, *p* = 0.27), but the StarVIBE showed a significant advantage detecting centrally located pulmonary nodules (66.7% vs. 16.7%, *p* = 0.031). There was a strong correlation in nodule size between CT and MRI sequences (HASTE: *ρ* = 0.80, *p* = 0.003; VIBE: *ρ* = 0.77, *p* < 0.001; StarVIBE: *ρ* = 0.78, *p* < 0.001). Mean image quality was rated as good to excellent for CT and MRI sequences.

**Conclusion:**

The overall lung nodule detection rate of StarVIBE was slightly, but not significantly, higher than conventional T1w VIBE and significantly higher than T2w HASTE. Detectability of centrally located nodules is better with StarVIBE than with VIBE. Nevertheless, all MRI analyses demonstrated considerably lower detection rates for small lung nodules, when compared to CT.

## Background

Magnetic resonance imaging (MRI) has made tremendous progress over the last decades, driven by new developments in sequence technique, reducing the overall examination time and improving image quality.

In oncological imaging, whole-body MRI (WB-MRI) has gained growing importance as a method for cancer staging and follow-up and is nowadays recommended in international guidelines of various tumor entities (e.g. multiple myeloma, prostate cancer, breast cancer) [1–3]. As MRI is a radiation-free imaging method, it is a valuable alternative to computed tomography (CT), especially in younger patients. One major limitation is its susceptibility to respiratory and cardiac motion resulting in a markedly reduced assessability of the lung parenchyma and limited detectability of potentially metastatic lung nodules compared with chest CT. Performing conventional MR imaging under breath-holding conditions is currently the common way to ensure a good image quality [4]. A strict immobility and compliance to breathing instructions is therefore required, which can sometimes be difficult in everyday clinical routine, especially when scanning children or multi-morbid older patients. The application of navigated schemes has been proposed, but is also prone to failure and leads to an extension of examination time [5]. Additionally, the use of fast MR sequences is required, which are associated with a loss of spatial image resolution [6].

The high susceptibility of conventional MR images to motion results from the line-by-line acquisition (Cartesian sampling) of the data space (k-space). Even small movements during the examination create disturbances in the phase encoding scheme and yield to phase offsets to the direction of the motion causing inconsistent phases in the k-space. This results in artifacts in the phase encoding direction [7, 8] in conventional MR sequences like T2-weighted HASTE (Half Fourier Acquisition Single shot Turbo spin Echo) and T1-weighted fat saturated post-contrast VIBE (Volume Interpolated Breath-Hold Examination) sequence, which are the most common sequences for lung nodule detection in MRI. These sequences are used for thoracic imaging, since they are fast and can be acquired slice-by-slice between breaths. The speed results from an incomplete filling or scanning of the k-space, which makes these sequences very susceptible for motion artifacts [9]. A possible solution to reduce the influence of motion is to change the way of k-space acquisition. A more recent example is the free-breathing radially acquired Stack of Stars T1-weighted gradient-echo (GRE) 3D VIBE sequence (StarVIBE) [6, 10]. In the StarVIBE sequence, data are acquired along individual radial spokes (Fig. 5). Due to the overlapping of the spokes in the center, phase errors can be reduced by averaging low frequency components. This overlap has a motion-averaging effect and consequently, images can be acquired during free-breathing, which not only reduces artifacts but also offers the advantage of a higher spatial resolution, since acquisition time is not limited to the duration of a breath hold [9].

The reduced influence of motion in chest MRI is particularly interesting in the assessment of lung cancer and pulmonary metastases, because MRI still has major disadvantages compared to CT for the reasons mentioned above. Whether this technology offers advantages in lung nodule detection compared to conventional MRI sequences has, to the best of our knowledge, not yet been investigated.

Thus, the purpose of our study was to compare the diagnostic potential of the StarVIBE sequence to the conventional T1-weighted fat saturated post-contrast VIBE sequence, the T2-weighted fat-suppressed HASTE sequence and to the reference standard CT for detection of pulmonary nodules in contrast-enhanced whole-body ^18^F-FDG PET/MRI.

## Material and methods

### Patients and inclusion criteria

The institutional review boards of the University Duisburg-Essen, Germany (study number 17–7396-BO) and Düsseldorf, Germany (study number 6040R) approved this study and it was performed in conformance with the Declaration of Helsinki [11]. After written informed consent was provided, a total of 88 women (mean age 52.5 ± 11.5 years, age range 28–82 years) with newly diagnosed therapy-naive breast cancer were prospectively enrolled in this study between April 2019 and April 2020. For cancer staging, all patients underwent a whole-body ^18^F-FDG PET/MRI as well as chest, abdomen and bone imaging recommended by current breast cancer guidelines, including thoraco-abdominal computed tomography, with a maximum of two weeks apart.

In accordance with the latest 2018 European Society For Medical Oncology (ESMO) guidelines for breast cancer [12], the following inclusion criteria had to be fulfilled to be eligible for whole-body staging: (1) newly diagnosed, treatment-naive T2-tumor or higher T-stage or (2) newly diagnosed, treatment-naive triple-negative tumor of every size or (3) newly diagnosed, treatment-naive tumor with molecular high risk (T1c, Ki67 > 14%, HER2-new over-expression, G3). Exclusion criteria were age < 18 years, medical contraindications to MRI or CT or contrast agents as well as former malignancies in the last 5 years, pregnancy, or breast-feeding.

### PET/MRI examination

All patients were instructed to fast for at least 6 h before the examination, and blood glucose levels were verified to be below 150 mg/dl. All patients underwent contrast-enhanced (Dotarem, Guerbet GmbH) ^18^F-FDG PET/MRI on an integrated 3.0-Tesla PET/MR system (Biograph mMR, Siemens Healthcare GmbH, Erlangen, Germany) with a mean delay of 76.6 ± 17.3 min after intravenous injection of a body weight adapted dosage of ^18^F-FDG (4 MBq/kg body-weight, mean activity: 240.9 ± 38.7 MBq). The scan volume covered head to the mid-thigh in supine position.

PET images were generated in four to five bed positions with a median of 3 min per bed position. PET data reconstruction was performed using an iterative 3D ordinary Poisson ordered-subset expectation maximization algorithm (3D OP-OSEM), 3 iterations and 21 subsets, a Gaussian filter with 4 mm Full Width at Half Maximum (FWHM) and a matrix dimension of 344 × 344 × 127 with an axial field of view (FOV) of 25.8 cm and a reconstructed image resolution of 2.09 × 2.09 × 2.03 mm.

For MR-based tissue attenuation correction (AC) and scatter correction the *syngo* MR E11P platform was used. A transaxial acquired high-resolution CAIPIRINHA (Controlled aliasing in parallel imaging results in higher spatial acceleration) T1-weighted three-dimensional (3D) Dixon-VIBE sequence was acquired, providing two sets of images (in- and opposed phase fat and water images) to generate a four-compartment (background air, lungs, fat, soft tissue) attenuation correction map (µmap) in coronal orientation. A bone atlas and truncation correction as proposed by Blumhagen et al. [13] were additionally applied [14–16]. MRI data were acquired simultaneously using a 16-channel head-and-neck radiofrequency (RF) coil, a 24-channel spine array RF coil, and 5 or 6-channel flex body coils, depending on patients’ height. Thoracic bed position was acquired with an acquisition time ranged from 6 to 8 min and with expiratory breath-hold, except for the duration of the 3D Stack of Stars GRE (StarVIBE) sequence, which was generated under free-breathing. Table 1 shows the scan parameters of the thoracic sequences.

**Table 1:** Scan parameters of the considered thoracic MRI sequences

Sequence	Orientation	Contrast agent	TA (s)	TE/TR (ms)	Slice thickness (mm)	Matrix size	FOV (mm^2^)
T2w HASTE	Axial	No	66	99/1.500	7.0	320 × 240	430 × 322
T1w fs VIBE	Axial	Yes	18	1.5/3.6	3	512 × 250	430 × 349
StarVIBE	Axial	Yes	220	1.5/3.2	1.1	520 × 204	360 × 360

T2w HASTE, T2-weighted half Fourier acquisition single shot turbo spin echo; T1w fs VIBE, T1-weighted fat-suppressed volume-interpolated breath-hold examination; StarVIBE, free-breathing 3D Stack of Stars GRE VIBE; TA, acquisition time; TR, repetition time; TE, echo time; FOV, field of view

### Computed tomography

In addition to the ^18^F-FDG PET/MRI all 88 patients underwent a thoraco-abdominal multi-slice contrast-enhanced (Ultravist 300™, Bayer Schering Pharma AG, Berlin, Germany) CT examination from skull base to the mid-thigh (Definition Edge or Definition Flash, Siemens Healthcare GmbH, Erlangen, Germany). The mean time between PET/MRI and CT scans were 3.1 ± 3.6 days (range 1–14 days). An automatic tube current modulation and automatic tube voltage selection was applied (CareDose 4D and CareKV, Siemens Heathcare GmbH, Erlangen). All scans were acquired in portal venous phase after intravenous application of a body-weight adapted dosage of non-ionic contrast agent. Thoracic images were reconstructed in lung window setting, using a sharp kernel (B70s) and a slice thickness of 2 mm.

### Image interpretation

The imaging datasets of CT (chest CT) and ^18^F-FDG PET/MRI (HASTE, VIBE, StarVIBE sequence and PET alone) were analyzed on a dedicated OsiriX workstation (Pixmeo, SARL, Bernex, Switzerland) and evaluated by 4 readers, two board-certified radiologists with 7 and 8 years of experience in hybrid imaging and two residents with 3 and 4 years of experience in hybrid imaging since graduating from medical school. CT and MRI datasets were assessed in random order and in separate sessions with at least two weeks apart to avoid recognition bias. Both readers were informed regarding the primary diagnosis of the patients but remained blinded to results of prior and follow-up imaging and to the patients’ history.

The quality of all thoracic imaging datasets was evaluated on a four-point Likert-scale (1 = very poor image quality: major artifacts; 2 = poor image quality: moderate artifacts; 3 = good image quality: minor artifacts; 4 = excellent image quality: no artifacts) and the presence and type of artifacts was documented. Lungs were systematically assessed in the same order, starting from the right upper lobe and continuing to the left lower lobe. Lung nodule number and location was noted, dividing the lungs into 4 quadrants (right upper, right lower, left upper, left lower) and subdividing lung sections into the regions pleural (lesions adjacent to the pleura), subpleural (within 1 cm of the pleura but not adjacent to it), and central (remaining lung tissue). Nodule size was measured (long-axis, short-axis and mean diameter) in millimeters. Furthermore, nodule contrast (1 = very low contrast; 2 = low contrast; 3 = moderate contrast; 4 = high contrast), nodule density (1 = solid; 2 = part-solid; 3 = pure ground glass) and nodule shape (1 = round; 2 = oval; 3 = ellipsoidal; 4 = lobular; 5 = notched; 6 = irregular) were documented. PET data in fused PET/MRI were evaluated noting presence of focal tracer uptake above background level (normal lung tissue), and for quantitative assessment SUVmax was measured by placing a polygonal volume of interest over each nodule.

### Statistical analysis

SPSS 24 (IBM, Armonk, NY, USA) was used for statistical analysis and all data are presented as mean ± standard deviation and median ± IQR. A *p* value < 0.05 was considered to indicate statistical significance. The Wilcoxon test was used to assess differences in nodule size, contrast, and image quality. For correlation analysis of nodule size, the Pearson’s correlation coefficient was applied. Because contrast is a categorical variable, the Spearman’s correlation coefficient was preferred.

## Results

### Nodule detection

**Fig. 1:**
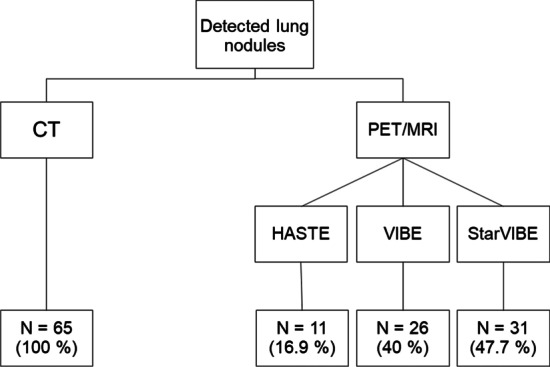
Flow-chart with numbers and percentages of detected lung nodules per MRI sequence, compared to CT

**Fig. 2:**
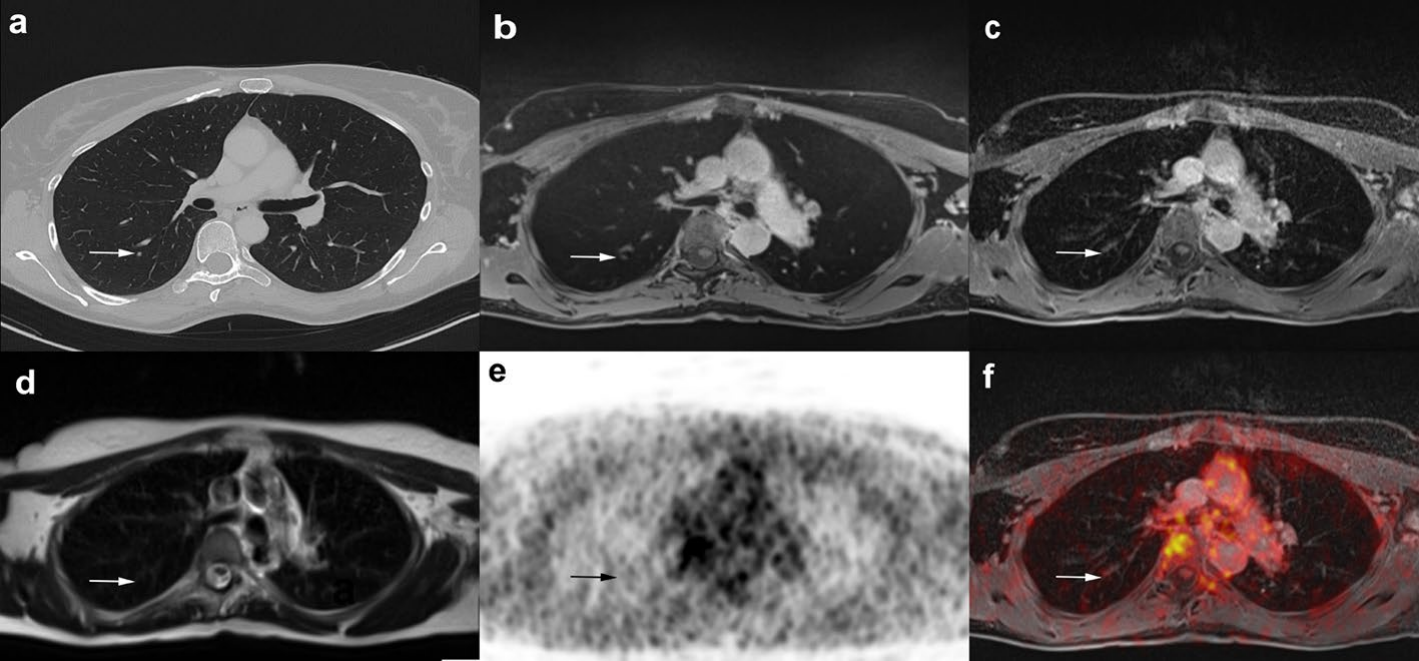
A 49-year-old female patient with histologically proven breast cancer. Four-millimeter centrally located lung nodule in the right lower lobe, which is clearly visible with CT (**a**) and the StarVIBE sequence (**b**). Caused by motion artifacts and lower resolution, the lung nodule was missed with the VIBE (**c**) and HASTE (**d**) sequence. No focal ^18^F-FDG uptake was seen on PET and fused ^18^F-FDG PET/MRI images (**e**, **f**)

According to the reference standard CT, there were 65 lung nodules in 36 of the 88 (40.9%) patients with a mean size of 3.7 ± 1.4 mm (range 2–8 mm, lung nodules per patient: 1–5, mean number 1.8 ± 1.6). In the remaining 52 (59.1%) patients, no lung nodules were present. Table 2 shows the distribution of lung nodules per patient. The HASTE sequence was able to detect 11 of 65 nodules (16.9%; 95% CI 8.8–28.3, mean size 4.1 ± 0.8 mm), while the post-contrast VIBE and the StarVIBE sequence detected 26 and 31 lung nodules (mean size 4.3 ± 1.4 mm and 3.9 ± 1.5 mm), respectively, resulting in detection rates of 40% (CI 28.0–52.9) and 47.7% (CI: 35.1–60.5) (Fig. 1). Detection rates between VIBE and StarVIBE did not differ significantly (difference 7.7%, 95% CI − 0.09 to 0.25, *p* = 0.27). There were no false positive results on MRI. Further results concerning detection rates between CT and the individual MRI sequences can be found in Table 3. The number of lung nodules was correctly identified by HASTE in 3 of 36 patients (8.3%), by VIBE in 10 of 36 patients (27.8%) and by the StarVIBE in 13 of 36 patients (36.1%). Compared to the VIBE, StarVIBE missed 4 subpleural nodules in 2 patients (mean size in CT 4.5 ± 0.58 mm), but detected additional 9 (3 subpleural, 6 central) primarily small nodules in 6 patients (mean size in CT 3.2 ± 0.97 mm) (Fig. 2). The HASTE sequence was able to detect one nodule that was not detected with VIBE or StarVIBE. This nodule was a calcified granuloma on CT. None of the morphologically visible and not-visible nodules showed an ^18^F-FDG uptake above the surrounding background level (mean SUVmax of all nodule detected with MRI sequences: 0.9 ± 0.5).

**Table 2:** Overview over the distribution of lung nodules among the patient collective

Number of patients	88
Patients with lung nodules	36
Patients without lung nodules	52
Lung nodules in total	65

Number of lung nodules per patient	Patients
1	19
2	9
3	5
4	2
5	1

**Table 3:** Comparison of the performance of CT with the individual MRI sequences and combined MRI for lung nodule detection

	Detected nodules	Relative difference (%)	95% confidence interval	*p* value
CT vs. HASTE	65 vs. 11	83.1	0.71 to 0.9	< 0.001
CT vs. VIBE	65 vs. 26	60	0.46 to 0.71	< 0.001
CT vs. StarVIBE	65 vs. 31	52.3	0.39 to 0.64	< 0.001
HASTE vs. VIBE	11 vs. 26	23.1	0.08 to 0.37	0.001
HASTE vs. StarVIBE	11 vs. 31	30.8	0.15 to 0.45	< 0.001
VIBE vs. StarVIBE	26 vs. 31	7.7	− 0.09 to 0.25	0.27
CT vs. MRI (combined)	65 vs. 36	44.6	0.31 to 0.57	< 0.001

The StarVIBE sequence was able to increase the overall lung nodule detection of MRI, but CT still showed a significantly better performance

### Comparison of nodule size and nodule contrast

The mean size of all lung nodules detected by CT was 3.7 ± 1.4 mm (range 2–8 mm, median 4.0 ± 1.5), compared to 4.1 ± 0.8 mm (range 2–5 mm, median 4.0 ± 1.0) by HASTE, 4.3 ± 1.4 mm (range 2–8 mm, median 4.0 ± 1.5) by VIBE, and 3.9 ± 1.5 mm (range 2–7 mm, median 4.0 ± 1.0) by StarVIBE. Analysis of size of corresponding nodule did not significantly differ between CT and MRI and between the MR sequences. Lung nodules missed by MRI were rather small, averaging 3.2 ± 1.3 mm on VIBE, 3.4 ± 1.4 mm on StarVIBE, and 3.6 ± 1.4 mm on HASTE, respectively (Fig. 2). A strong correlation in nodule size between CT and HASTE (*r* = 0.80, *p* = 0.003), VIBE (*r* = 0.77, *p* < 0.001), and StarVIBE (*r* = 0.78, *p* < 0.001) as well as between StarVIBE and VIBE (*r* = 0.94, *p* < 0.001) and StarVIBE and HASTE (*r* = 0.81, *p* < 0.02) could be observed. Nodule contrast differed significantly between CT and MRI in corresponding nodules, rated as moderate in CT with a mean of 2.91 ± 0.79 (median 3.0 ± 1.0), poor in StarVIBE (mean 2.07 ± 0.98, median 2.0 ± 2.0, *p* = 0.001) and VIBE (mean 2.00 ± 0.75, median 2.0 ± 0.25, *p* < 0.001), and very poor in the HASTE sequence (mean 1.45 ± 0.52, median 1.0 ± 1.0, *p* = 0.004). There was no statistically significant correlation between CT and MRI in nodule contrast (HASTE: *ρ* = 0.15, *p* = 0.67; VIBE: *ρ* = 0.17, *p* = 0.42; StarVIBE: *ρ* = 0.18, *p* = 0.34).

### Localization

**Fig. 3:**
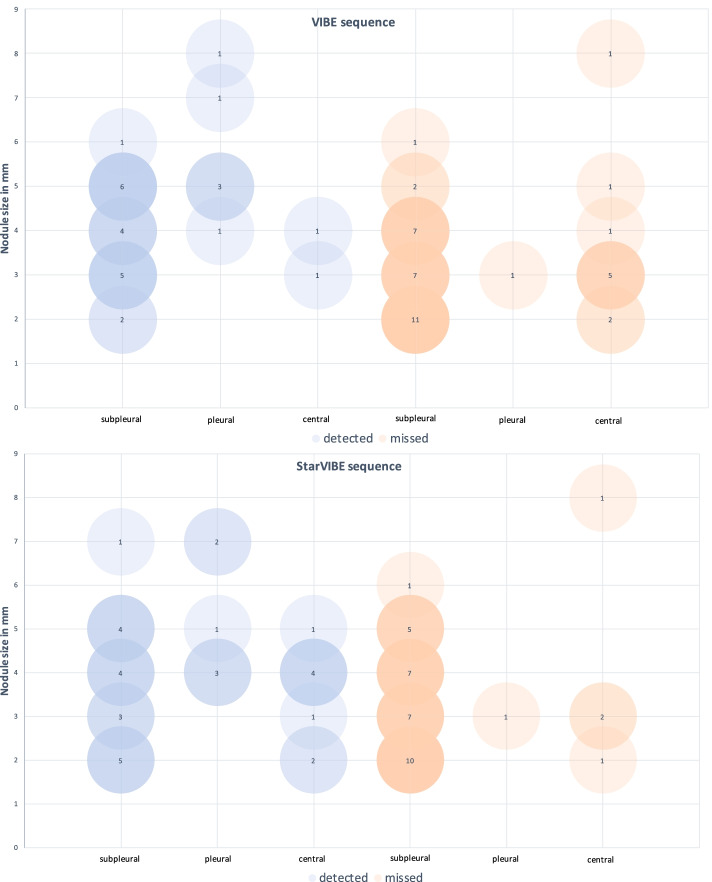
Bubble chart of detected and missed lung nodules on VIBE (upper chart) and StarVIBE (lower chart). The lung nodules are subdivided according to their size and localization. The number of lung nodules of one size is represented with color intensity of the bubbles and their labeling. For the missed nodules, the size was taken from the reference standard CT. The missed lung nodules were rather small in VIBE (mean 3.2 ± 1.3 mm vs. 4.3 ± 1.4 mm) and StarVIBE (mean 3.4 ± 1.4 mm vs. 3.9 ± 1.5 mm)

Forty-two of the 65 (64.6%) detected lung nodules were located in the lower parts of the lungs and 23 (36.4%) in the upper parts. Seven lung nodules were located adjacent to the pleura, while 46 were positioned subpleurally and 12 centrally in the lungs. The local distribution of missed lung nodules showed significant differences between VIBE and StarVIBE particularly in the detection of centrally located nodules (Table 4, 16.7% vs 66.7%, difference 50.0%, 95% CI 10.8–75.4, *p* = 0.031) (Figs. 2, 3).

**Table 4:** Distribution of detected and missed nodules according to their location in the lung for each sequence

	CT	HASTE		VIBE		StarVIBE	
	Detected	Detected	Missed	Detected	Missed	Detected	Missed
Adjacent to pleura	7	2 (29.6%)	5 (71.4%)	6 (85.7%)	1 (14.3%)	6 (85.7%)	1 (14.3%)
Subpleural	46	8 (17.4%)	38 (82.6%)	18 (39.1%)	28 (60.9%)	17 (37.0%)	29 (63.0%)
Central	12	1 (8.3%)	11 (91.7%)	2 (16.7%)	10 (83.3%)	8 (66.7%)	4 (33.3%)

### Image quality

**Fig. 4:**
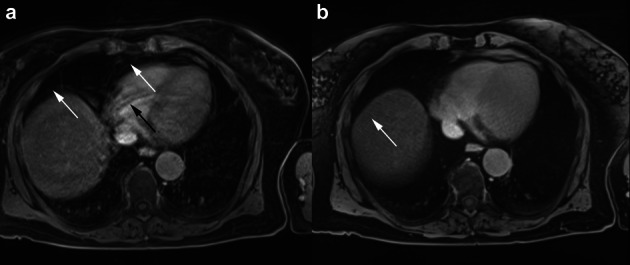
Comparison of VIBE (**a**) and StarVIBE (**b**) sequence in a 70-year-old female patient with histologically proven breast cancer. Lower lung section with parts of the diaphragm and heart. Limited Image quality of the VIBE sequence due to respiratory artifacts, heart motion (**a** white arrows) and ghosting (**a** black arrow). In the StarVIBE sequence minimal streak artifacts are visible (**b** white arrow), which have only a mild effect on image quality

The mean image quality was rated as good to excellent for both CT and all MRI sequences. Nevertheless, the statistical analysis yielded differences between CT (mean 3.99 ± 0.09, median 4.0 ± 0.0) and MRI (HASTE: mean 3.91 ± 0.29, median 4.0 ± 0.0, *p* < 0.001; VIBE: mean 3.60 ± 0.68, median 4.0 ± 1.0, *p* < 0.01; StarVIBE: mean 3.91 ± 0.35, median 4.0 ± 0.0, *p* < 0.001). The difference between VIBE and StarVIBE also yielded a statistical significance with a better performance of the StarVIBE sequence (*p* < 0.01). Almost no artifacts were documented in CT imaging, while in 30 of 88 patients (34.0%) artifacts were documented in VIBE images, especially due to respiratory and cardiac motion and less also aliasing and ghosting. The StarVibe images were only affected by streak artifacts (14/88, 15.9%), which hardly influenced the assessability of lung nodules (Table 5, Fig. 4).

**Table 5:** Number of documented artifacts in CT and the individual MRI sequences

	CT	HASTE	VIBE	StarVIBE
Respiratory/cardiac motion	2	12	23	
Aliasing		2	7	
Ghosting			3	
Streak artifacts				14

The VIBE sequence in particular proved to be susceptible to breathing and heart motion artifacts, while the StarVIBE was only influenced by streak artifacts, which had only a minor impact on the image quality. CT was only hardly influenced by artifacts

## Discussion

In this study, we investigated the potential of the free-breathing Stack of Stars GRE StarVIBE sequence for the detection of lung nodules in breast cancer patients and compared it with the performance of CT and other common MRI sequences using ^18^F-FDG PET/MRI. Accurate and reliable detection of lung nodules is an important aspect of image-based cancer staging, since the identification of a metastatic spread has a considerable influence on treatment and ultimately on patient’s survival, regardless of the cancer entity [17–19]. CT is still the gold standard, because of its clear superiority in the detection of even very small pulmonary nodules [20, 21]. While MRI has been shown to be equivalent for the detection of nodules > 10 mm, the capability of detecting smaller lung nodules is limited [22–24]. Even though most small nodules < 10 mm in oncologic patients are known to be benign (i.e. post-infectious, indurative etc.), about 20% of those lung nodules represent early metastases, with a high chance to be missed by chest MRI, even when using state-of-the-art T1-weighted breath hold GRE, pulse sequences or Propeller/Blade sequences (Periodically Rotated Overlapping Parallel Lines with Enhanced Reconstruction) [23, 25, 26]. Main reasons are a low tissue density in the lungs, rapid signal loss at the transition from lung to soft tissue, and artifacts caused by cardiac and respiratory motion [7]. Currently, T1-weighted gradient echo or pulse sequences such as VIBE with a short echo time offer a reasonable result and are therefore recommended for MR-based lung nodule detection [27–29]. However, more sensitive MRI sequences have to be developed to increase the acceptance of MRI as a valuable thoracic staging examination [27].

One way to improve the detection of lung nodules by MRI is to change the way of k-space acquisition. In radial sampling, the k-space data are acquired along radial spokes. This was already used in the past for example in the Propeller/Blade sequences. The basic idea here is to use a set of radially directed strips or "blades", which are rotating around the k-space center. Each blade is composed of multiple parallel phase-encoded lines that can be collected using fast spin echo or gradient echo methods.

**Fig. 5:**
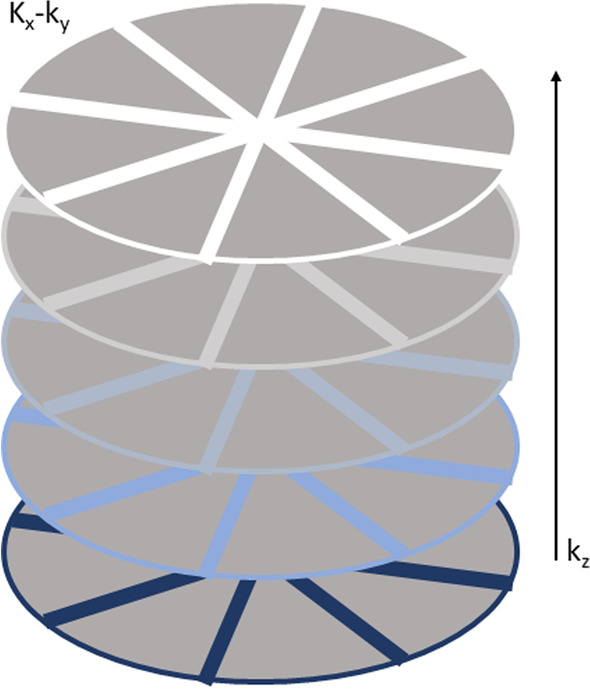
“Stack of Stars” approach. Acquisition of the in-plane dimension (*k*_*x*_ − *k*_*y*_) along radial spokes, while in slice direction (*k*_*y*_) the conventional Cartesian sampling is applied, resulting in a cylindrical coverage. Inspired by [9]

3D Stack of Stars approach represents the actual radial sampling, means applying the recently developed motion robust free-breathing radial T1-weighted gradient-echo 3D VIBE sequence for the acquisition of the in-plane dimension (kx-ky) along individual radial lines without rotation, while in slice direction the conventional sampling is applied, resulting in a cylindrical coverage [7, 9, 30] (Fig. 5). This allows a further reduction of motion artifacts even with free breathing and a consistent high spatial resolution. Consequently, the reduction of motion-related artifacts may improve the assessment of motion-prone organs, especially the lung.

Most studies dealing with this topic differentiate between sensitivity rates for MRI concerning nodules less or more than 10 mm in size. For nodules greater than 10 mm, various studies have shown that MRI sequences have a similar detection rate to CT and, as a combined PET/MRI examination, can also provide information on nodule dignity [23, 24, 27, 30–32]. In concordance with the results of prior studies for smaller lung nodules [20, 21, 27, 30, 32], CT outperformed the currently used MRI sequences in our study, which offered detection rates of 40% (VIBE) and 16.9% (HASTE). The StarVIBE sequence was even able to detect 47.7%, but also remained clearly inferior to CT. In a study by Sawicki et al. [27], comparing lung nodule detection of ^18^F-FDG PET/MRI and ^18^F-FDG PET/CT in 121 oncologic patients, nodules smaller than 5 mm were found with the VIBE sequence in 43.1%. Furthermore, Rauscher et al. [32] described significantly lower detection rates for nodules < 10 mm when comparing PET/CT and PET/MRI in the detection of 40 lung nodules with a detection rate of 45.5% for the VIBE sequence. Chandarana et al. [30] compared ^18^F-FDG PET/MRI and ^18^F-FDG PET/CT in the detection of lung nodules using a radially acquired VIBE sequence, a preclinical prototype of the StarVIBE sequence. In their study, the radial VIBE received a sensitivity rate of 62.3% of all lung nodules (43/69), but only 28% (7/25) of the nodules < 5 mm were detected [30]. In keeping with their results, the mean size of missed lung nodules was 3.23 ± 1.31 mm and 3.41 ± 1.42 for VIBE and StarVIBE, so especially small nodules < 4 mm were missed (Fig. 2). The rather good results of the StarVIBE sequence result primarily from the fact, that images can be acquired in free-breathing, which is particularly important for elderly people with comorbidities or children, who are often staged using MRI, trying to keep the radiation burden as low as possible. Even though the acquisition of individual StarVIBE slices takes longer than of conventional MR sequences, a major advantage of performing exams during free-breathing is continuous data acquisition, resulting in sharper images and improved resolution (here, 1.1 mm isotropic). Although StarVIBE has the smallest voxel volume compared to the others, it still missed slightly larger nodules than VIBE did (3.42 mm vs. 3.23 mm). This could be, because radial imaging tends to have image blurring when motion or magnetic susceptibility differences are present. Additionally, because StarVIBE acquires the k-space lines in z direction rectilinearly, it may still have small aliasing artifacts in this direction.

When comparing the localization of detected and missed lung nodules in the VIBE and StarVIBE sequence, no relevant differences between pleural and subpleural nodules were found. However, the StarVIBE sequence detected significantly more centrally located lung nodules. Presumably, the detection of central, perihilar nodules is particularly hampered by heart motion and breathing and the lower susceptibility to artifacts of the StarVIBE sequence has a direct effect on the detection rate of pulmonary nodules.

In our study, none of the lung nodules presented a focal tracer uptake on the PET component. Yet, in various studies [33–35], it has been shown that PET negativity is not suitable to exclude malignancy, especially in lung nodules smaller than 10 mm, since there is a high proportion of false-negative PET diagnoses caused by motion- and breathing artifact and the limited spatial resolution of the PET component, which can lead to an underestimation of the true FDG avidity [26, 36, 37].

CT provided a significantly better contrast of lung nodules than MRI sequences. This was primarily due to the high number of granulomas, which were mostly small in size and frequently showing partial calcification. While CT offers a strong contrast between such high-density structures and aerated lungs, many lung nodules remain invisible in MRI due to low-proton density and susceptibility artifacts of the calcified parts.

This study is not without limitations. Only women with newly diagnosed breast cancer were enrolled, who were generally younger and physically better constituted than most other people suffering from cancer. Commonly, older, multi-morbid patients tend to have problems holding their breath and lying still, hence, the benefits of StarVIBE might be more significant in such cohorts. Furthermore, there might have been an influence of the different slice thicknesses in CT and MRI sequences and the additional evaluation of coronal and sagittal CT reconstructions for lung nodule detection. However, the slice thicknesses and plane directions used in this study were clinical standard and thus our results should be valid to represent the performance of CT and MRI for lung nodule detection in everyday clinical routine. Moreover, the mean time between PET/MRI and CT scans were 3 days (range 1–14 days), hence interim intra-individual changes in size or number of lung nodules, while not likely, cannot totally be excluded. Overall, it is a relatively small cohort of patients, even though it is one of the largest studies of its kind. Further research in this field with larger cohorts is needed in the future.

In conclusion, this study has demonstrated that lung nodule detection with the free-breathing T1-weighted 3D Stack of Stars GRE (StarVIBE) MR sequence as part of a whole-body ^18^F-FDG PET/MRI cancer staging protocol was not significantly higher than with conventional breath-hold T1-weighted VIBE and significantly higher than with T2-weighted HASTE. The StarVIBE sequence seems to be especially advantageous in detecting centrally located lung nodules. However, PET/MRI still has a substantially limited sensitivity in lung nodule detection compared to CT and thus bears the risk of missing small lung metastases in oncologic patients. Further research towards more sensitive MRI sequences is necessary.

## Data Availability

The datasets used and/or analysed during the current study are available from the corresponding author on reasonable request.

## References

[1] Gradishar WJ, Anderson BO, Balassanian R, Blair SL, Burstein HJ, Cyr A (2018). Breast cancer, Version 4.2017, NCCN clinical practice guidelines in oncology. J Natl Compr Cancer Netw..

[2] Kumar SK, Callander NS, Hillengass J, Liedtke M, Baljevic M, Campagnaro E (2019). NCCN guidelines insights: multiple myeloma, Version 1.2020. J Natl Compr Cancer Netw..

[3] Mottet N, Bellmunt J, Bolla M, Briers E, Cumberbatch MG, De Santis M (2017). EAU-ESTRO-SIOG guidelines on prostate cancer. Part 1: screening, diagnosis, and local treatment with curative intent. Eur Urol..

[4] Schafer JF, Vollmar J, Schick F, Seemann MD, Kamm P, Erdtmann B (2005). Detection of pulmonary nodules with breath-hold magnetic resonance imaging in comparison with computed tomography. Rofo.

[5] Bruegel M, Gaa J, Woertler K, Ganter C, Waldt S, Hillerer C (2007). MRI of the lung: value of different turbo spin-echo, single-shot turbo spin-echo, and 3D gradient-echo pulse sequences for the detection of pulmonary metastases. J Magn Reson Imaging.

[6] Block KT, Chandarana H, Milla S, Bruno M, Mulholland T, Fatterpekar G (2014). Towards routine clinical use of radial stack-of-stars 3D gradient-echo sequences for reducing motion Sensitivity. J Korean Soc Magn Reson Med.

[7] Kumar S, Rai R, Stemmer A, Josan S, Holloway L, Vinod S (2017). Feasibility of free breathing Lung MRi for radiotherapy using non-Cartesian k-space acquisition schemes. Br J Radiol.

[8] McRobbie DW, Moore EA, Graves MJ, Prince MR (2017). MRI from picture to proton.

[9] Block KT, Chandarana H, Fatterpekar G, Hagiwara M, Milla S, Mulholland T (2013). Improving the robustness of clinical T1-weighted MRI using radial VIBE. Magnetom Flash..

[10] Azevedo RM, De Campos ROP, Ramalho M, Herédia V, Dale BM, Semelka RC (2011). Free-breathing 3D T1-weighted gradient-echo sequence with radial data sampling in abdominal MRI: preliminary observations. Am J Roentgenol.

[11] World Medical Association Declaration of Helsinki (2013). ethical principles for medical research involving human subjects. JAMA.

[12] Cardoso F, Senkus E, Costa A, Papadopoulos E, Aapro M, Andre F (2018). 4th ESO-ESMO international consensus guidelines for advanced breast cancer (ABC 4)dagger. Ann Oncol Off J Eur Soc Med Oncol.

[13] Blumhagen JO, Ladebeck R, Fenchel M, Scheffler K (2013). MR-based field-of-view extension in MR/PET: B0 homogenization using gradient enhancement (HUGE). Magn Reson Med.

[14] Paulus DH, Quick HH, Geppert C, Fenchel M, Zhan Y, Hermosillo G (2015). Whole-body PET/MR imaging: quantitative evaluation of a novel model-based MR attenuation correction method including bone. J Nucl Med.

[15] Lindemann ME, Oehmigen M, Blumhagen JO, Gratz M, Quick HH (2017). MR-based truncation and attenuation correction in integrated PET/MR hybrid imaging using HUGE with continuous table motion. Med Phys.

[16] Oehmigen M, Lindemann ME, Gratz M, Kirchner J, Ruhlmann V, Umutlu L (2018). Impact of improved attenuation correction featuring a bone atlas and truncation correction on PET quantification in whole-body PET/MR. Eur J Nucl Med Mol Imaging.

[17] Barth A, Wanek LA, Morton DL (1995). Prognostic factors in 1,521 melanoma patients with distant metastases. J Am Coll Surg.

[18] Biederer J, Beer M, Hirsch W, Wild J, Fabel M, Puderbach M (2012). MRI of the lung (2/3). Why... when ... how?. Insights Imaging..

[19] AJCC Cancer Staging Manual (2017). AJCC cancer staging manual.

[20] Sommer G, Koenigkam-Santos M, Biederer J, Puderbach M (2014). Role of MRI for detection and characterization of pulmonary nodules. Radiologe.

[21] Biederer J, Hintze C, Fabel M (2008). MRI of pulmonary nodules: technique and diagnostic value. Cancer Imaging.

[22] Sawicki LM, Grueneisen J, Schaarschmidt BM, Buchbender C, Nagarajah J, Umutlu L (2016). Evaluation of 18F-FDG PET/MRI, 18F-FDG PET/CT, MRI, and CT in whole-body staging of recurrent breast cancer. Eur J Radiol.

[23] de Galiza BF, Geismar JH, Delso G, Messerli M, Huellner M, Stolzmann P (2018). Pulmonary nodule detection in oncological patients—value of respiratory-triggered, periodically rotated overlapping parallel T2-weighted imaging evaluated with PET/CT-MR. Eur J Radiol.

[24] Biondetti P, Vangel MG, Lahoud RM, Furtado FS, Rosen BR, Groshar D (2021). PET/MRI assessment of lung nodules in primary abdominal malignancies: sensitivity and outcome analysis. Eur J Nucl Med Mol Imaging.

[25] Benjamin MS, Drucker EA, McLoud TC, Shepard JAO (2003). Small pulmonary nodules: detection at chest CT and outcome. Radiology.

[26] Sawicki LM, Grueneisen J, Buchbender C, Schaarschmidt BM, Gomez B, Ruhlmann V (2016). Evaluation of the outcome of lung nodules missed on 18F-FDG PET/MRI compared with 18F-FDG PET/CT in patients with known malignancies. J Nucl Med.

[27] Sawicki LM, Grueneisen J, Buchbender C, Schaarschmidt BM, Gomez B, Ruhlmann V (2016). Comparative performance of 18F-FDG PET/MRI and 18F-FDG PET/CT in detection and characterization of pulmonary lesions in 121 oncologic patients. J Nucl Med.

[28] Regier M, Kandel S, Kaul MG, Hoffmann B, Ittrich H, Bansmann PM (2007). Detection of small pulmonary nodules in high-field MR at 3 T: evaluation of different pulse sequences using porcine lung explants. Eur Radiol.

[29] Schäfer JF, Vollmar J, Schick F, Seemann MD, Kamm P, Erdtmann B (2005). Detection of pulmonary nodules with breath-hold magnetic resonance imaging in comparison with computed tomography. Rofo.

[30] Chandarana H, Heacock L, Rakheja R, DeMello LR, Bonavita J, Block TK (2013). Pulmonary nodules in patients with primary malignancy: comparison of hybrid PET/MR and PET/CT imaging. Radiology.

[31] Dewes P, Frellesen C, Al-Butmeh F, Albrecht MH, Scholtz JE, Metzger SC (2016). Comparative evaluation of non-contrast CAIPIRINHA-VIBE 3T-MRI and multidetector CT for detection of pulmonary nodules: in vivo evaluation of diagnostic accuracy and image quality. Eur J Radiol.

[32] Rauscher I, Eiber M, Fürst S, Souvatzoglou M, Nekolla SG, Ziegler SI (2014). PET/MR imaging in the detection and characterization of pulmonary lesions: technical and diagnostic evaluation in comparison to PET/CT. J Nucl Med.

[33] Yilmaz F, Tastekin G (2015). Sensitivity of (18)F-FDG PET in evaluation of solitary pulmonary nodules. Int J Clin Exp Med.

[34] Khalaf M, Abdel-Nabi H, Baker J, Shao Y, Lamonica D, Gona J (2008). Relation between nodule size and 18F-FDG-PET SUV for malignant and benign pulmonary nodules. J Hematol Oncol.

[35] Farid K, Poullias X, Alifano M, Regnard J-F, Servois V, Caillat-Vigneron N (2015). Respiratory-gated imaging in metabolic evaluation of small solitary pulmonary nodules: 18F-FDG PET/CT and correlation with histology. Nucl Med Commun.

[36] Bar-Shalom R, Valdivia AY, Blaufox MD (2000). PET imaging in oncology. Semin Nucl Med.

[37] Cruickshank A, Stieler G, Ameer F (2019). Evaluation of the solitary pulmonary nodule. Intern Med J.

